# Phosphoethanolamine Modification of *Neisseria gonorrhoeae* Lipid A Reduces Autophagy Flux in Macrophages

**DOI:** 10.1371/journal.pone.0144347

**Published:** 2015-12-07

**Authors:** Susu M. Zughaier, Justin L. Kandler, Jacqueline T. Balthazar, William M. Shafer

**Affiliations:** 1 Laboratory of Bacterial Pathogenesis, Department of Veterans Affairs Medical Center Decatur, Atlanta, Georgia, United States of America; 2 Department of Microbiology and Immunology, Emory University School of Medicine, Atlanta, Georgia, United States of America; 3 Emory Antibiotic Resistance Center, Emory University School of Medicine, Atlanta, Georgia, United States of America; University of Alabama at Birmingham, UNITED STATES

## Abstract

Autophagy, an ancient homeostasis mechanism for macromolecule degradation, performs an important role in host defense by facilitating pathogen elimination. To counteract this host defense strategy, bacterial pathogens have evolved a variety of mechanisms to avoid or otherwise dysregulate autophagy by phagocytic cells so as to enhance their survival during infection. *Neisseria gonorrhoeae* is a strictly human pathogen that causes the sexually transmitted infection, gonorrhea. Phosphoethanolamine (PEA) addition to the 4' position of the lipid A (PEA-lipid A) moiety of the lipooligosaccharide (LOS) produced by gonococci performs a critical role in this pathogen’s ability to evade innate defenses by conferring decreased susceptibility to cationic antimicrobial (or host-defense) peptides, complement-mediated killing by human serum and intraleukocytic killing by human neutrophils compared to strains lacking this PEA decoration. Heretofore, however, it was not known if gonococci can evade autophagy and if so, whether PEA-lipid A contributes to this ability. Accordingly, by using murine macrophages and human macrophage-like phagocytic cell lines we investigated if PEA decoration of gonococcal lipid A modulates autophagy formation. We report that infection with PEA-lipid A-producing gonococci significantly reduced autophagy flux in murine and human macrophages and enhanced gonococcal survival during their association with macrophages compared to a PEA-deficient lipid A mutant. Our results provide further evidence that PEA-lipid A produced by gonococci is a critical component in the ability of this human pathogen to evade host defenses.

## Introduction

*Neisseria gonorrhoeae* (hereafter termed Gc) is a strict human pathogen that causes the sexually transmitted infection termed gonorrhea. Gc causes more than 100 million new cases of gonorrhea each year as estimated by the World Health Organization [1]. With the emergence of antibiotic-resistant Gc strains and the prediction that gonorrhea may become an untreatable disease as a consequence, the CDC recently listed Gc as a pathogen with an “urgent threat” to public health [2,3,4]. Symptomatic gonococcal infections of the genitourinary tract in males and females are the result of a significant pro-inflammatory response characterized by purulent exudates with a significant presence of polymorphonuclear leukocytes (PMNs) with infected PMNs containing viable Gc. Importantly, lower genital tract gonococcal infections in females are often asymptomatic, but if left untreated can lead to pelvic inflammatory disease, ectopic pregnancy, and infertility [5].

A hallmark of Gc pathogenesis is the ability of this pathogen to survive a multitude of innate antimicrobial host defenses that become available at mucosal surfaces or in bodily fluids during infection. In addition to resisting the oxidative and non-oxidative killing systems of PMNs, Gc can display resistance to serum complement and antimicrobial compounds that bathe mucosal surfaces such as cationic antimicrobial peptides [6], fatty acids [7], and progesterone [8]. Gc can also modulate metabolic processes of infected host cells that might otherwise be detrimental to bacterial survival. For example, we recently reported that Gc modulate the host iron-limiting innate immune defenses in macrophages to facilitate intracellular survival [9]. Further, Bergman et al reported that Gc down-regulate expression of the CAMP LL-37 in cervical epithelial cells as another mechanism of immune evasion [10].

**Fig 1:**
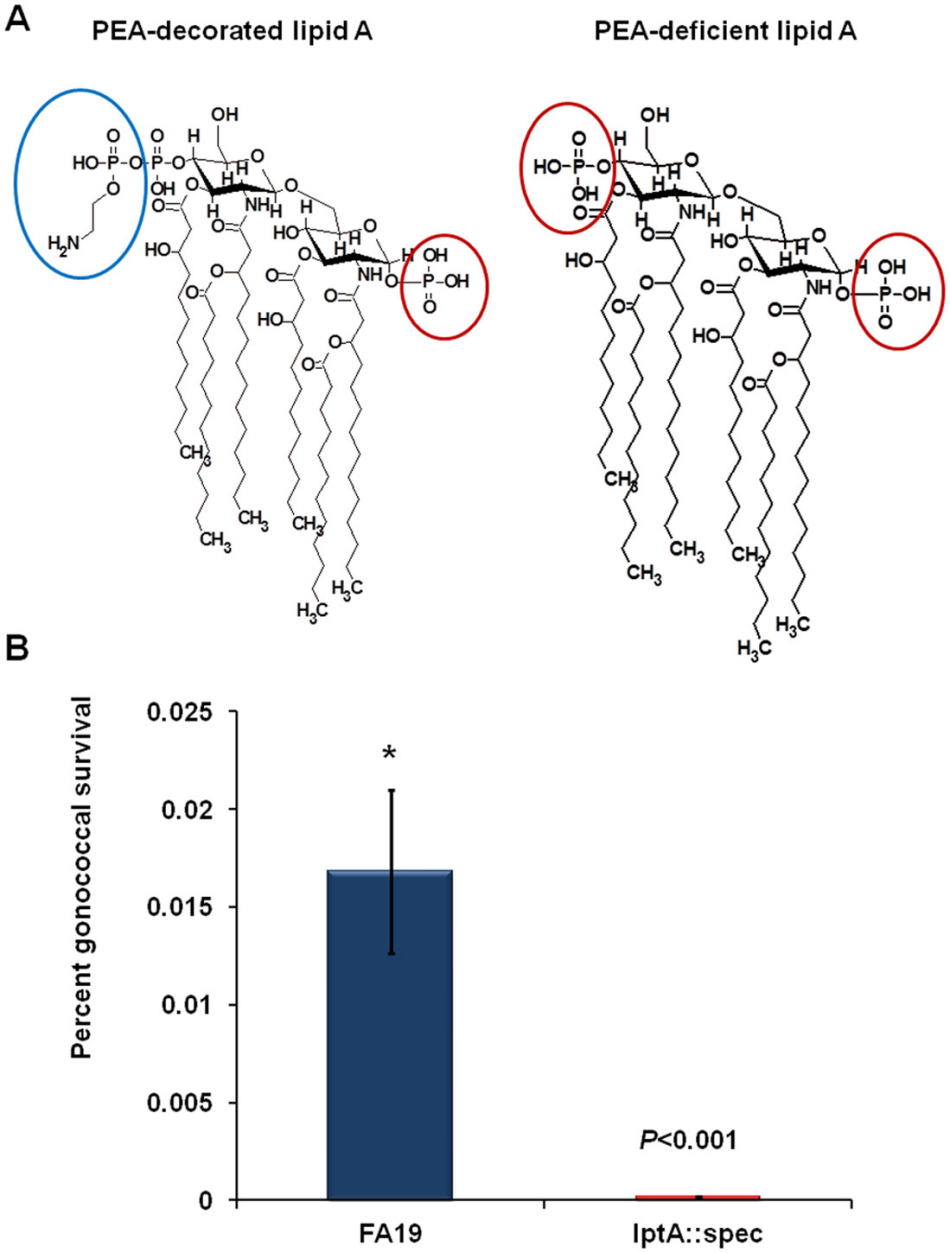
PEA modification of Gc lipid A enhances Gc survival during association with murine macrophages. (A): Schematic presentation of the lipid A structures of Gc strains with or without PEA modification. The PEA moiety containing a positively charged amino group is circled in blue and negatively charged phosphate groups are circled in red. PEA is absent from the lipid A of the *lptA*::*spec* mutant [16]. (): Percent survival of the total Gc inoculum in association with RAW264 macrophage culture was assessed after 2 hrs of phagocytosis. Murine RAW264 macrophages were infected with live WT (FA19) or mutant (*lptA*::*spec*) at an MOI of 50 (n = 3). After one hour of phagocytosis, macrophages were washed extensively to remove extracellular Gc that are not tightly bound to cells. Survival of Gc associated with macrophages was assessed after 2 hrs of phagocytosis using standard plating and CFU counts. *p* values were calculated using Student *t*-test in reference to cells infected with the WT (*) strain. These data are representative of three independent experiments.

Recently, phosphoethanolamine (PEA) modification of lipid A has been shown to be important for Gc resistance to innate host defenses, bacterial fitness during experimental lower genital tract infection of female mice or human male volunteers [11], and the ability of this pathogen to stimulate a pro-inflammatory response. PEA is added to the 4' position of lipid A by a PEA transferase, which is encoded by the phase variable *lptA* gene. This decoration adds a positive charge to the lipid A head group (Fig 1), thereby decreasing binding of CAMPs [16, 17] to the Gc surface resulting in decreased susceptibility to CAMPs. More recently, Handing and Criss [15] showed that PEA-lipid A can enhance Gc resistance to killing by human PMNs likely as a result of conferring decreased bacterial susceptibility to lysosomal cationic antimicrobial proteins known to have anti-Gc action (e.g., cathepsin G) [18]; this modification has also been implicated in delaying fusion of azurophilic granules with maturing phagolysosomes [19]. Furthermore, PEA modification of Gc lipid A modulates surface binding of C4b binding protein, thus providing resistance to complement-mediated killing by the classical pathway [20].

Given the strong association of Gc PEA-lipid A with bacterial resistance to innate host defenses, modulation of intraleukocytic metabolic events and Gc fitness during infection, we examined if this structure would also impact the fate of Gc-infected host cells. As a model to test this possibility we examined if Gc infection of macrophages influences autophagy. Autophagy (“self-eating”) is a physiological process important for cell survival and is required for normal cellular homeostasis, recycling macromolecules, and is activated upon starvation [21, 22]. Importantly, autophagy plays an important role in host defense by clearing invading pathogens [23, 24]. Autophagy is complex and requires a set of specific proteins that interact in an orchestrated manner to remove and recycle macromolecules from the cytosol by directing the cargo to the lysosomal degradation pathway [25]. The autophagy process is initiated by the formation of cytosolic, double-membraned vesicles that become elongated and envelop macromolecules (or bacteria in the case of infection) and then fuse with lysosomes to complete the degradation of the enclosed contents. The process of fusing an autophagosomal vesicle with a lysosomal vesicle is referred to as “autophagic flux”, which if inhibited leads to defective or impaired autophagy [26].

Defective autophagy is associated with chronic maladies such as Huntington’s disease [27, 28], Crohn’s disease [29, 30], and cystic fibrosis [31, 32]. Additionally, bacteria-mediated dysregulation of autophagy has been associated with exacerbation of acute infections. In order to facilitate their survival and evade clearance by the host, some bacterial pathogens evolved systems to manipulate the autophagy process using different mechanisms (reviewed in detail by Huang and Brumell) [23]. Thus, pathogens may impede autophagy at different steps including the inhibition of cellular signaling pathways required for initiating the autophagy process [33, 34], direct interference with the activity of autophagy components [35, 36], inhibition of autophagic flux by blocking autophagosome-lysosome fusion [37–40], utilizing autophagy vesicle resources for bacterial replication [41–43], escaping from the autophagosome to the cytosol [44–46], and evading autophagy recognition by masking bacterial surfaces [47–51].

Since PEA modification of lipid A increases Gc fitness in the host, and has important effects on the host innate immune response to gonococcal infection, we hypothesized that this lipid A modification may contribute to Gc survival through evasion of autophagy within infected host innate immune cells. We now provide evidence that Gc producing PEA-lipid A not only have a survival advantage when associated with phagocytes, but that they most likely also have an enhanced ability to dysregulate autophagy in infected macrophages and can influence host cell chemokine production. Taken together, the present work and past studies [11, 12, 14, 16] implicate Gc PEA-lipid A as a Gc virulence factor.

## Materials and Methods

### Reagents

RPMI 1640 medium, Dulbecco’s modified Eagle medium (D-MEM), fetal bovine serum (FBS), penicillin/streptomycin, sodium pyruvate and non-essential amino acids were obtained from Cellgro Mediatech (Herdon, VA). Human and murine CXCL10 (IP-10) and CXCL3 (MIP-2β) ELISA kits were from R&D Systems (Minneapolis, MN). Cyto-ID^®^ autophagy Detection Kit was purchased from Enzo Life Sciences (Farmingdale, NY). Eritoran [52] [53], a TLR4 inhibitor was a gift from Eisai Inc. (Andover MA,USA).

### Bacterial culture conditions

Gc strain FA19 (wild-type; WT), its isogenic mutant *lptA*::*spec* strain and complemented (*lptA C’*: a functional *lptA* gene is expressed in the mutant *lptA*::*spec* to restore its function), strains were previously described [16]. These Gc strains were grown as pilus-positive, opacity-negative colony variants on GC agar containing defined Supplements I and II and 1 mM isopropyl β-D-1-thiogalactopyranoside (IPTG) under 5.0% (v/v) CO_2_ at 37°C as described by Shafer et al. [54]. Broth cultures of gonococci were grown in GC broth with supplements and 0.043% (w/v) sodium bicarbonate at 37°C in a shaking water bath; in this media WT FA19 strain and its isogenic mutant *lptA*::*spec* have similar growth rates (data not presented). The viability of Gc cultures was determined using dilution plating onto GC agar and colony forming units (CFU) were enumerated after 24 hr of incubation at 37°C in a CO_2_ incubator. Gc grown on agar plates were resuspended in unsupplemented GC broth and harvested by centrifugation at 5,000x*g* for 10 minutes. The bacterial pellet was washed twice with PBS and resuspended in 10 ml of D-MEM tissue culture medium without antibiotics to prepare a live Gc inoculum for macrophage infection experiments (see below) [9].

### Cell cultures

THP-1 human macrophage-like monocytic cell line was obtained from the American Type Culture Collection (ATCC, Manassas, VA) and grown in RPMI 1640 with L-glutamate supplemented with 10% (v/v) FBS, 50 IU/ml of penicillin and 50 μg/ml of streptomycin. Culture flasks were incubated at 37°C with humidity and 5% (v/v) CO_2_. Murine macrophages (RAW264 cell line from ATCC) were grown in D-MEM supplemented and incubated as noted above.

### Macrophage infection assay

Freshly grown human THP-1 macrophage-like monocytic cells (in the absence of antibiotics) were adjusted to one million cells/ml, then transferred into 8-well tissue culture plates (2 ml/well) and infected with live Gc FA19, FA19 *lptA*::*spec*, or the complemented strain (FA19 *lptA C’*) at an MOI (multiplicity of infection) of 50 then incubated overnight at 37°C with 5% (v/v) CO_2_. Uninfected cells in triplicate wells were also incubated simultaneously and were used as a minus infection control. Supernatants from infected or uninfected macrophages were harvested and saved at -20°C for determination of chemokines release and cells were washed with PBS, pelleted (1000 x *g* for 5 min), and saved at -80°C for western blot analysis. In parallel experiments, cells were washed and stained using the Cyto-ID^®^ autophagy Detection Kit (Enzo Life Sciences, Farmingdale, NY) following the manufacturer’s instructions in order to monitor autophagic flux. Briefly, infected and uninfected THP-1 cells (at 1 million cells/ml) were washed twice with 1 ml of diluted assay buffer, then resuspended in 100 μl of Microscopy Dual Detection Reagent Solution (prepared by adding 4 μl of Cyto-ID^®^ Green Detection Reagent and 1 μl of Hoechst 33342 nuclear stain to 1 ml of diluted assay buffer supplemented with 5% [v/v] FBS). Cell pellets were gently resuspended and incubated for 30 min at 37°C in the dark. Cells were then washed twice with diluted assay buffer and resuspended in 100 μl of the same assay buffer. One drop (~40 μl) of stained cells was placed on a clean microscope glass slide, overlaid with a coverslip, and sealed immediately. Autophagic flux in stained cells was monitored within 30 min using fluorescence microscopy (Olympus IX8S1F-3, Olympus Corporation, Japan) at 60x magnification and selecting the standard FITC filter for imaging autophagic flux and the DAPI filter for nuclear staining. Images from at least 10 different fields were collected and analyzed for autophagic flux index, which was calculated by counting the number of green fluorescent puncta in each cell in multiple fields and dividing by the total number of cells in those same fields as determined by counting DAPI-stained nuclei.

### Autophagy induction and visualization by confocal microscopy

To investigate whether PEA-lipid A impacts autophagy, the RAW264 macrophage cell line stably transfected with a construct encoding the autophagy marker GFP-LC3 (a kind gift from Dr. Alfred Merrill, Georgia Institute of Technology, Atlanta, GA) was used [55]. LC3 (microtubule-associated light chain 3) is a cytosolic protein and a specific constituent of the autophagosomal membrane [56]. Upon autophagosome formation, GFP-LC3 is recruited and co-localized to the autophagosome membrane; this process can be visualized by fluorescence microscopy as the formation of fluorescent puncta [57]. Briefly, freshly grown murine RAW264 macrophages were harvested by scraping and adjusted to 1 million cells/ml and seeded on pre-cleaned glass cover slips (24x24 #1 from Surgipath Medical Industries Inc.) placed in 8-well tissue culture plates and incubated overnight. Adherent macrophages were washed twice with PBS and placed in antibiotic-free DMEM medium containing 10% heat-inactivated FBS prior to infection with FA19, FA19 *lptA*::*spec*, or FA19 *lptA C’* strains at an MOI of 50 followed by incubation at 37°C overnight with 5% CO_2_. Macrophages were washed three times with PBS and fixed with 4% paraformaldehyde before staining with nuclear stain DAPI for 5 min at room temperature. Glass cover slips were inverted and mounted on glass slides and sealed immediately. Autophagy induction was monitored using laser scanning confocal microscopy (Olympus IX8S1F-3, Olympus Corporation, Japan). Multiple fields (n = 16) were examined in each individual glass cover slip and images were captured and analyzed using FV10-ASW software from Olympus. Autophagosome formation was quantified based on the fluorescence intensity of GFP-LC3 positive autophagic vacuoles [57] as described previously [58]. Briefly, the autophagy index was calculated using image analysis software FV10-ASW that systematically defined regions of interest (ROI) across all fields and the intensity of GFP-LC3 fluorescence was divided by the intensity of blue DAPI stain that reflects the number of cells per field. Further, autophagy induction was manually quantitated by counting the number of GFP-LC3 puncta in each field divided by the number of cells. Since LPS/LOS induces TLR4 activation which contributes to autophagy induction, in some experiments, Eritoran, a TLR4 inhibitor [59] was used at 5μg/ml to block TLR4 activation during macrophage infection with Gc strains. As a control, highly purified LOS [60] from wild type gonococci strain FA19 that expresses PEA-lipid A [16], which was kindly provided by R. Carlson (Complex Carbohydrate Center, University of Georgia, Athens, Georgia) was used at 50 ng/ml dose with or without Eritoran (5 μg/ml), incubated overnight and autophagy induction was monitored as above.

### Macrophage bactericidal assay

To determine whether PEA-lipid A plays a role in survival of Gc in association with macrophages as was seen by Handing and Criss for human PMNs [15], we employed murine RAW264 macrophage bactericidal assays as previously described [9]. Briefly, freshly grown Gc strains were adjusted to an OD600 of 1.0 (~1x10^8^ CFU/ml) in antibiotic-free D-MEM medium containing 10% heat-inactivated fetal bovine serum (FBS). Macrophages were also freshly grown, washed and adjusted to 1 million cells/ml in antibiotic-free D-MEM medium containing 10% FBS. Since these RAW264 macrophages are adherent, cells were seeded in 24-well tissue culture plate (1 million cells/well) and allowed to adhere overnight prior to infection with live gonococci at an MOI of 50 as described above. After one hour of initiated phagocytosis at 37°C, adherent RAW264 cells were washed three times with antibiotic-free medium containing 10% heat-inactivated FBS and all fluids were carefully removed without disturbing the adherent macrophages. One ml of fresh antibiotic-free medium containing 10% heat-inactivated FBS was added to each well and infected cells were further incubated for 1 hr. Extracellular Gc were removed by washing adherent macrophages three times with DMEM medium. Viable intracellular (or tightly adherent) Gc were assessed by serial plating of macrophage cultures lysed using 0.01% triton X-100 in PBS as previously described [9].

### Chemokine release quantification

The released CXCL10 (IP-10) and CXCL3 (MIP2β) in supernatants from RAW264 macrophages infected with live Gc were quantified by DuoSet ELISA (R&D Systems, Minneapolis, MN) as previously described [61].

### Statistical analysis

Mean values ± SD (standard deviation) and *p* values (Student *t-* test) of at least three independent determinations were calculated with Microsoft Excel software.

## Results and Discussion

### Phosphoethanolamine modification of Gc lipid A enhances bacterial survival and reduces autophagosome formation in murine macrophages

The presence of pathogens triggers innate host defenses and autophagy, as an antibacterial process, is induced to facilitate pathogen clearance and control of infection. However, bacteria have evolved a variety of mechanisms to evade recognition by the autophagy process. We previously reported that *N*. *gonorrhoeae* survives in association with human and murine monocytes and macrophages and responds to an iron-limited environment [9]. Given recent reports regarding the role of Gc PEA-lipid A in determining bacterial resistance to innate host defenses [12, 15, 16] and as a contributor to fitness during experimental infections in mice and men [11], we investigated whether this lipid A decoration plays a role in enhancing bacterial survival and autophagy evasion. For this purpose, we first infected murine macrophages with isogenic Gc differing in the presence of PEA-lipid A (Fig 1A) and examined whether PEA-lipid A (Fig 1A) facilitates survival in association with macrophages as reported recently for survival of Gc in human PMNs [15]. The viability of Gc strains during infection was determined using macrophage bactericidal assays as previously described [9]. We observed that the gonococcal *lptA*::*spec* mutant was killed more rapidly in a macrophage bactericidal assay when compared to the PEA-lipid A producing WT strain FA19 (Fig 1B). These data are consistent with previous reports showing that Gc lacking PEA-lipid A are, compared to the PEA-lipid A positive parent strain, less fit during experimental infection of female mice and human males and are cleared more rapidly in these models of lower genital tract infection [11].

**Fig 2:**
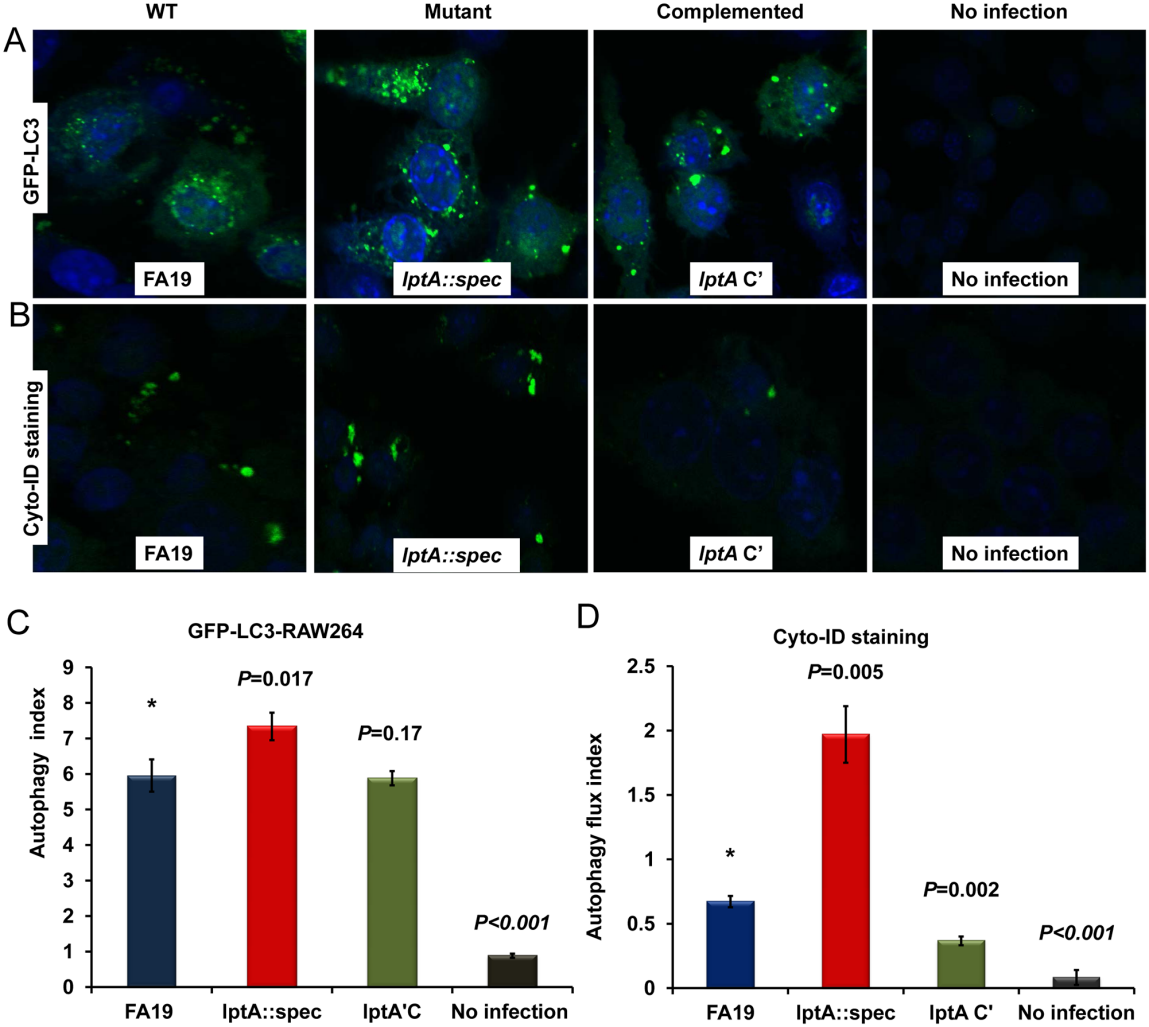
PEA modification of Gc lipid A reduces autophagosome formation in infected murine macrophages. (A): Representative images of autophagy induction in RAW264 macrophages stably transfected with the marker of autophagy GFP-LC3 was visualized by confocal imaging. RAW264 cells were infected with live WT (FA19), mutant (*lptA*::*spec*) or the complemented (*lptA* C') gonococcal strains at an MOI of 50. (B): Representative confocal microscopic images of autophagic flux in RAW264 macrophages stained with Cyto-ID^®^ autophagy reagent, which partitions specifically into the autophagolysosomal vesicles, indicating active autophagy induction. RAW264 macrophages were infected with live gonococci as in panel A. (C): Autophagy index was calculated using image analysis software FV10-ASW that systematically defined regions of interest (ROI) across all fields (n = 16) and the intensity of GFP-LC3 fluorescence was divided by the intensity of blue DAPI stain that reflects the number of cells per field. Error bars represent the ±SD from the mean of autophagy index of different fields. (D): Autophagic flux index was calculated by counting green puncta and dividing by the number of cells (DAPI-stained blue nuclei) in multiple fields. Error bars represent the ±SD from the mean of autophagic flux index of 8 different fields. *p* values were calculated using Student *t*-test in reference to cells infected with the WT (*) strain. These data are representative of three independent experiments.

We then investigated whether this lipid A decoration plays a role in autophagy evasion. Autophagy induction was monitored by measuring fluorescence of the marker of autophagy LC3 in murine RAW264 macrophages stably transfected with a GFP-tagged LC3 construct using confocal imaging [55]. We found that the Gc lacking PEA-lipid A (FA19 *lptA*::*spec*) induced significantly more autophagy in RAW264 macrophages compared to the WT or complemented strains producing PEA-lipid A (Fig 2A). In contrast, the uninfected macrophages induced minimal autophagy consistent with the basal level of induction during cellular homeostasis (Fig 2A). The autophagy index was calculated using image analysis software FV10-ASW that systematically defined regions of interest (ROI) across all fields and the intensity of GFP-LC3 fluorescence was divided by the intensity of blue DAPI stain that reflects the number of cells per field (Fig 2C). Similar results were obtained when autophagy induction was manually quantitated by counting the number of GFP-LC3 puncta in each field divided by the number of cells (data not shown).

The fusion of an autophagosome with a lysosome (known as “autophagic flux”) is necessary for the completion of the autophagy process and the consequent degradation of macromolecular/bacterial contents. In order to confirm that the visualized GFP-LC3 puncta induced by Gc infection represents active autophagic flux and not just arrested autophagosomes, we used the Cyto-ID^®^ autophagy staining probe that selectively labels autophagic vacuoles and specifically partitions into the lysosomal compartment [62, 63]. Autophagic flux was confirmed in murine RAW264 macrophages, devoid of any GFP-tagged marker, when infected with the test strains at an MOI 50 overnight. The results (Fig 2B) showed that the *lptA*::*spec* strain lacking PEA-lipid A induced significantly more autophagic flux compared to the WT or complemented strains. The uninfected cells did not show any significant Cyto-ID^®^ autophagy staining indicating that the probe specifically stained autophagolysosomes confirming active autophagic flux (Fig 2D). As a control for autophagic flux, RAW264 macrophages were treated with 20 μM of chloroquine (CQ) with or without infection with WT FA19 gonococci. CQ is a lysosomotropic agent that prevents lysosome acidification which in turn inhibits degradation and recycling of the phagolysosome cargo, consequently leading to the accumulation of LC3 in the autophagic vacuoles that can be monitored with the Cyto-ID^®^ autophagy probe [64]. CQ treatment alone without Gc infection did not enhance visualization of autophagic flux. However, when CQ treatment was combined with gonococcal infection in RAW264 macrophages it enhanced visualization of autophagic flux (S1 Fig), suggesting that LC3II is accumulating and gonococci may be targeted to autophagic vacuoles. Taken together, these data show that PEA modification of Gc lipid A reduces autophagic flux in macrophages and also confirm that PEA-lipid A is necessary for survival of Gc when associated with phagocytes.

**Fig 3:**
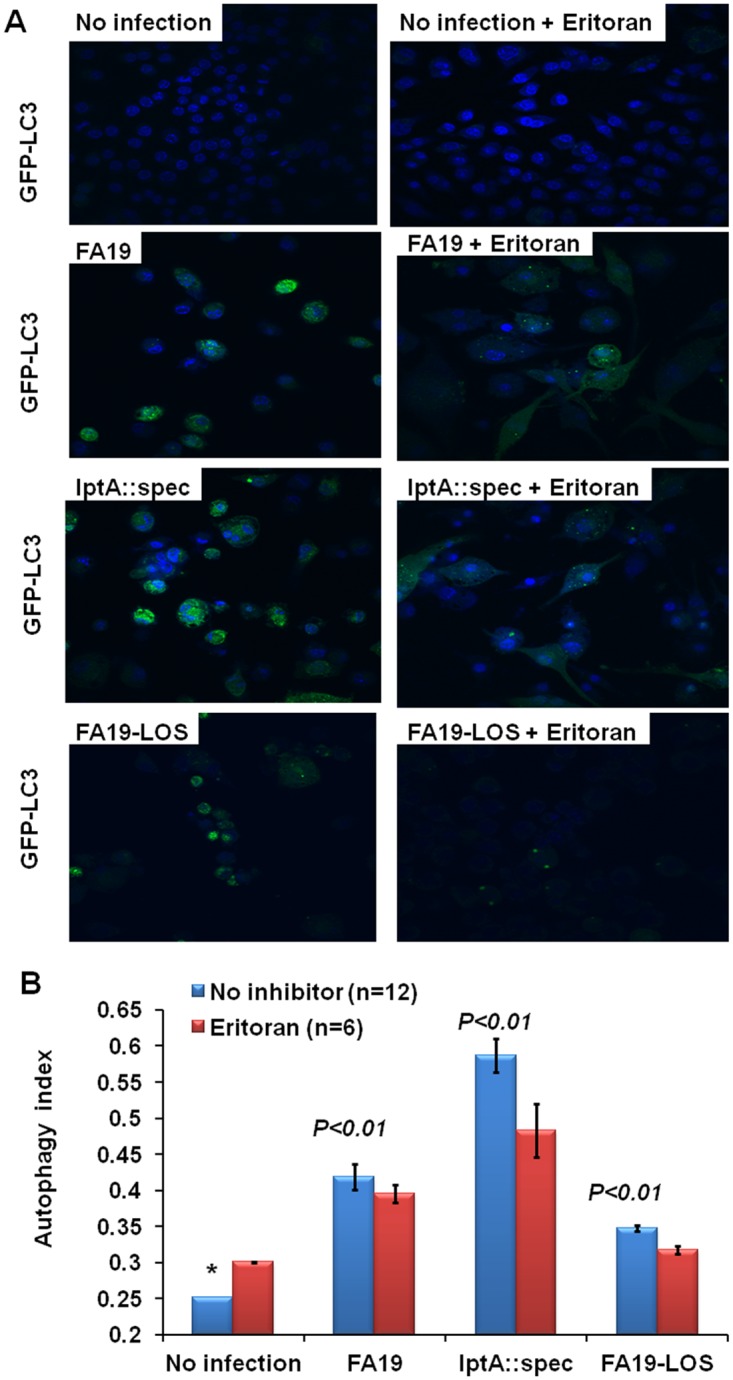
TLR4 contributes to autophagy induction in murine macrophages infected with live Gc. (A): Representative confocal microscopic images of autophagic puncta formation in GFP-LC3-tagged RAW264 macrophages infected with live Gc strains FA19 or FA19 *lptA*::*spec* at an MOI of 50, or with purified WT (FA19) gonococcal LOS (50 ng/ml) with and without Eritoran (5 μg/ml) treatment. (B): Autophagy index was calculated using image analysis software FV10-ASW that systematically defined regions of interest (ROI) across all fields and the intensity of GFP-LC3 fluorescence was divided by the intensity of blue DAPI stain that reflects the number of cells per field. Error bars represent the ±SEM from the mean. *p* values were calculated using Student *t*-test in reference to cells infected with the WT (*) strain. These data are representative of two independent experiments.

Since TLR4, the LPS receptor, is a sensor for inducing autophagy as an innate immune response [57], we examined the contribution of TLR4 in autophagy induction during Gc infection in macrophages. To this end, we used Eritoran, a TLR4 inhibitor [52] [53] [65] to inhibit TLR4-mediated autophagy induction during live infection with Gc strains. Eritoran is a synthetic hypo-acylated lipid A antagonist molecule that inhibits TLR4 activation by binding to MD-2 (the TLR4 co-receptor) in place of LPS/LOS, consequently preventing TLR4 receptor complex multimerization [66]. We also examined autophagy induction using highly purified LOS from WT Gc strain FA19 that express PEA-lipid A. As expected, the data show that autophagy induction in murine RAW264 macrophages infected with Gc strains FA19 and the FA19 *lptA*::*spec* mutant was reduced in presence of 5 μg/ml of Eritoran (Fig 3). Similarly, Eritoran inhibited autophagy induction when gonococcal LOS (50 ng/ml) was used to induce autophagy (Fig 3). Thus, the data suggest that LOS contributes to autophagy induction via TLR4 signaling. In support of this observation, highly purified LOS with PEA-deficient lipid A from *Neisseria meningitidis* [17] also induced more autophagy in macrophages when compared to WT LOS from meningococcal PEA-sufficient lipid A (Zughaier, unpublished). Therefore, we propose that the PEA-lipid A decoration performs an important role in modulating autophagy induction in macrophages during neisserial infection.

### Gonococci producing PEA-lipid A have an increased capacity to dysregulate autophagy in human phagocytes

**Fig 4:**
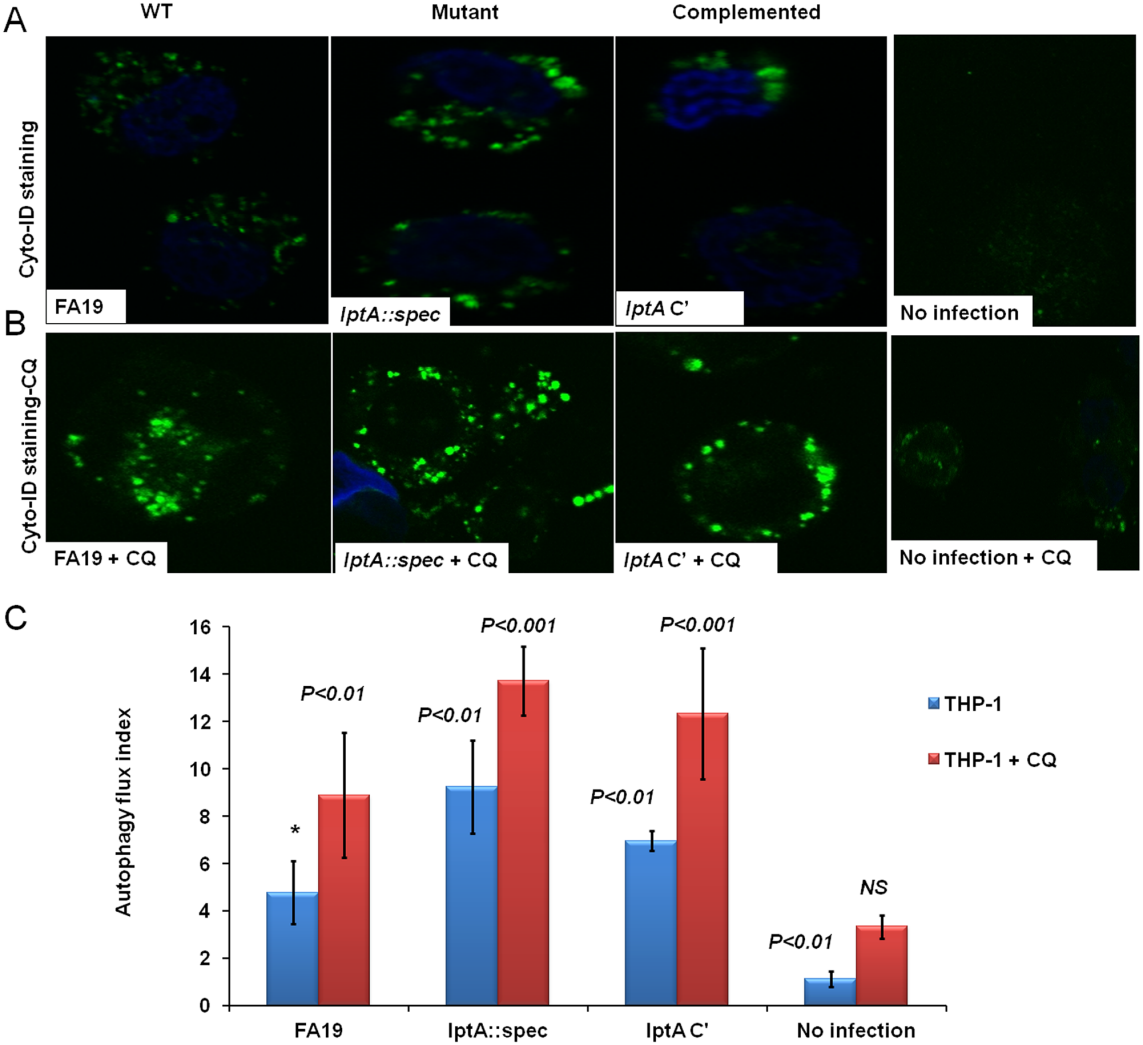
Autophagic flux in human THP-1 cells infected with live Gc. PEA modification of Gc lipid A reduces autophagic flux in THP-1 cells. (A): Representative confocal microscopic images (from different fields) of autophagic flux in THP-1 cells stained with Cyto-ID^®^ autophagy probe, which partitions specifically into the autophagolysosomal vesicles, indicating active autophagy induction. THP-1 cells were infected with Gc strains at an MOI of 50. (B): Representative confocal microscopic images of autophagic flux in THP-1 cells treated with 20 μM chloroquine (CQ), infected with live Gc and stained with Cyto-ID^®^ autophagy probe. **C**: Autophagic flux index was calculated by counting green puncta divided by the number of cells (DAPI-stained blue nuclei) in multiple fields. Error bars represent the ±SD from the mean of autophagic flux index of 8 different fields. *p* values were calculated using Student *t*-test in reference to cells infected with the WT FA19 (*) strain without CQ. These data are representative of two independent experiments performed in technical duplicates.

Since Gc is a strictly human pathogen, we also infected human macrophage-like monocytes (THP-1 cells) with the isogenic strains differing in PEA-lipid A and evaluated autophagy. THP-1 cells (devoid of any GFP-tagged construct) were infected as described above prior to staining with Cyto-ID^®^ autophagy probe and autophagic flux was quantitated using confocal microscopy. In agreement with the results seen in Fig 2, *lptA*::*spec* Gc lacking PEA-lipid A induced significantly more autophagic flux in THP-1 cells compared to the WT or the complemented strains, whereas the uninfected THP-1 cells did not show any significant staining (Fig 4A). As a control for autophagic flux, THP-1 cells were treated with 20 μM of chloroquine (CQ) with or without infection with live Gc (Fig 4B). As expected, CQ treatment combined with gonococcal infection in THP-1 cells enhanced fluorescence of autophagic vacuoles (Fig 4C) suggesting that LC3 is accumulating and gonococci may be targeted to autophagic vacuoles. During autophagy, the autophagy marker LC3I becomes lipidated to form LC3II, which then inverts or localizes inside of the autophagosome reflecting active autophagy typically visualized as puncta [26]. Thus, our data suggest that lack of PEA modification significantly increased puncta formation in macrophages (Fig 4A and 4B) reflecting more autophagy induction.

### PEA modification of gonococcal lipid A impacts chemokine release from infected macrophages

Macrophages play an important role in host defense by sensing the presence of pathogens and respond by initiating an array of innate immune responses to help slow and clear the infection. Infected macrophages secrete large quantities of inflammatory mediators that help recruit leukocytes, mainly neutrophils, to the site of infection [67]. We and others reported that infection with Gc leads to a substantial release of cytokines and chemokines from macrophages and other phagocytes [9, 68–70]. Recently, using our test strains Packiam et al. reported that Gc lacking PEA decoration of lipid A induced a significantly dampened vaginal pro-inflammatory cytokine and chemokine response during murine experimental genital tract infection compared to PEA-lipid A producing strains [12, 13]. Since induction of autophagy reduces cytokine and chemokine release from activated macrophages [71, 72], we used chemokine release as a marker to ascertain whether PEA decoration of Gc lipid A impacts release of important immune biological signals from macrophages at sites of infection. For this purpose, we measured the release of macrophage chemokines such as inflammatory protein-2 β (MIP-2β), also known as CXCL3, and interferon gamma-inducible small protein 10 (IP-10), also known as CXCL10, from RAW264 macrophages infected with the panel of Gc strains differing in PEA-lipid A at an MOI of 50. MIP-2β controls migration and adhesion of monocytes and macrophages and exerts chemotaxis activity for macrophages and neutrophils [73]. Similarly, IP-10 is rapidly and massively induced in macrophages upon sensing LPS/LOS and exerts leukocyte chemotaxis activity to recruit macrophages, neutrophils and T lymphocytes [74, 75], therefore playing a role in linking innate and adaptive immunity. As expected, infection with gonococci induced a large increase in RAW264 macrophage chemokine production compared to uninfected controls (S2 Fig). Furthermore, RAW264 macrophages infected with Gc having PEA-lipid A showed a small (~1.3 fold), but significant (*p* = 0.002) increase in the release of MIP-2β and IP-10 compared to the *lptA*::*spec* mutant. These data are in agreement with previous studies showing that lack of PEA-lipid A reduces release of proinflammatory mediators and recruitment of leukocytes [12, 70].

PEA modification of Gc lipid A is now recognized as an important property of this pathogen to evade host defenses [12,13,15,16,17]. We propose that PEA-lipid A enhances the ability of Gc to delay or otherwise dysregulate autophagy by infected host phagocytic cells. Delaying clearance of infected phagocytes by autophagy is now recognized as a strategy used by pathogens to promote their survival and possible dissemination during infection. The mechanisms of autophagy dysregulation by pathogens are complex and varied (21). For example, *Salmonella typhimurium* [34], *Vibrio cholera* [76], *Mycobacterium tuberculosis* [77] and *Bacillus anthracis* [76] are reported to inhibit cellular signaling required to initiate the autophagy process. Other pathogens like *Yersinia pestis* [40], *Helicobacter pylori* [78, 79], *Mycobacterium* species [37, 80] and *Chlamydia trachomatis* [39] evade the autophagy process by blocking the fusion of the autophagosome with the lysosome. Several bacterial pathogens such as *Legionella pneumophila* [81], *Brucella melitensis* [82], *Coxiella burnettii* [42], *Anaplasma phagocytophilum* [41] and *Staphylococcus aureus* [83] are reported to manipulate and hijack autophagy for replication and intracellular survival.

The mechanism by which PEA-lipid A enhances the ability of Gc to delay or otherwise dysregulate autophagy of infected mouse or human macrophages is not clear. As shown in Fig 3, treatment with the TLR4 antagonist Eritoran reduced the autophagy index most prominently in macrophages infected with PEA-deficient gonococci. Since the anionic charges of naked lipid A phosphate groups are important for binding to cationic amino acid residues in TLR4 and for hydrogen bonding with MD2 [see Figure 2D in Park et al [66]; The structural basis of lipopolysaccharide recognition by the TLR4-MD-2 complex], perhaps TLR4-mediated induction of autophagic flux is modulated by PEA-deficient lipid A due to its greater potential for ionic bonding between LOS and the TLR4-MD2 complex. It is also interesting to note that PEA is a precursor in the synthesis of phosphatidylethanolamine (PE), the major phospholipid component of cellular membranes. In the process of autophagy, the cytosolic LC3I protein is lipidated by covalent conjugation to PE in the autophagic membrane to form LC3II which co-localizes within the mature double-membraned autophagolysosome. Specifically, the LC3I carboxyl terminal glycine forms an amide linkage with the PEA head group of PE to facilitate LC3-PE tethering in the membrane [26]. This lipidation step is essential for autophagosome formation since compromised LC3I lipidation is reported to inhibit autophagosome maturation and is thus an autophagy evasion strategy [36]. For example, Choy et al. reported that *Legionella pneumophila* directly subverts autophagy using its bacterial effector RavZ to inhibit LC3-PE conjugation [36]. It is therefore plausible to consider that PEA-lipid A produced by Gc might interfere with the LC3I lipidation step by acting as a decoy or molecular mimic, thus reducing LC3II tethering to autophagolysosome and consequently reducing autophagy. Despite the uncertainty of the mechanism by which PEA-lipid A influences autophagy by Gc-infected phagocytes, we suggest that this property would allow gonococci to persist and possibly migrate from infected sites during infection.

## Supporting Information

S1 Fig Chloroquine treatment enhances autophagy flux in murine macrophages infected with live Gc. Since Cyto-ID^®^ autophagy probe partitions specifically into the autophagolysosomal vesicles indicating active autophagy flux, chloroquine (CQ) is used as a control for autophagy flux. (A): Representative confocal microscopic images of autophagic puntca formation in GFP-LC3-tagged RAW264 macrophages infected with live Gc strain FA19 at an MOI of 50 with and without CQ (20 μM) treatment. (B): Representative confocal microscopic images of autophagic flux in murine RAW264 macrophages treated with 20 μM CQ, infected with live Gc strain FA19 and stained with Cyto-ID^®^ autophagy probe.
(TIF)

S2 Fig PEA modification of Gc lipid A impacts chemokine release from infected macrophages. Murine RAW264 macrophages infected with Gc strains at an MOI of 50. Chemokines MIP-2β (A) and IP-10 (B) release from infected macrophages was quantitated by ELISA. Error bars represent the ±SD from the mean chemokine release from three independent biological replicates. *p* values were calculated using Student *t*-test in reference to results obtained when cells were infected with the WT FA19 (*) strain.
(TIF)

## Abbreviations

Gc: gonococci or *Neisseria gonorrhoeae*
LptA: lipid A phosphoethanolamine transferase
LOS: lipooligosaccharide
CFU: colony forming unit
CAMP: cationic antimicrobial peptide
LC3: microtubule-associated light chain 3
GFP: green fluorescent protein
PEA: phosphoethanolamine
PE: phosphatidylethanolamine
MIP-2β: chemokine inflammatory protein-2 β (synonym CXCL3)
IP-10: interferon gamma-inducible small protein 10 (synonym CXCL10)
