# Supplementary figures and images for: Phosphoethanolamine Modification of Neisseria gonorrhoeae Lipid A Reduces Autophagy Flux in Macrophages
Source: PLoS One. 2015 Dec 7;10(12):e0144347. doi: 10.1371/journal.pone.0144347 (PMC4671640; doi:10.1371/journal.pone.0144347)

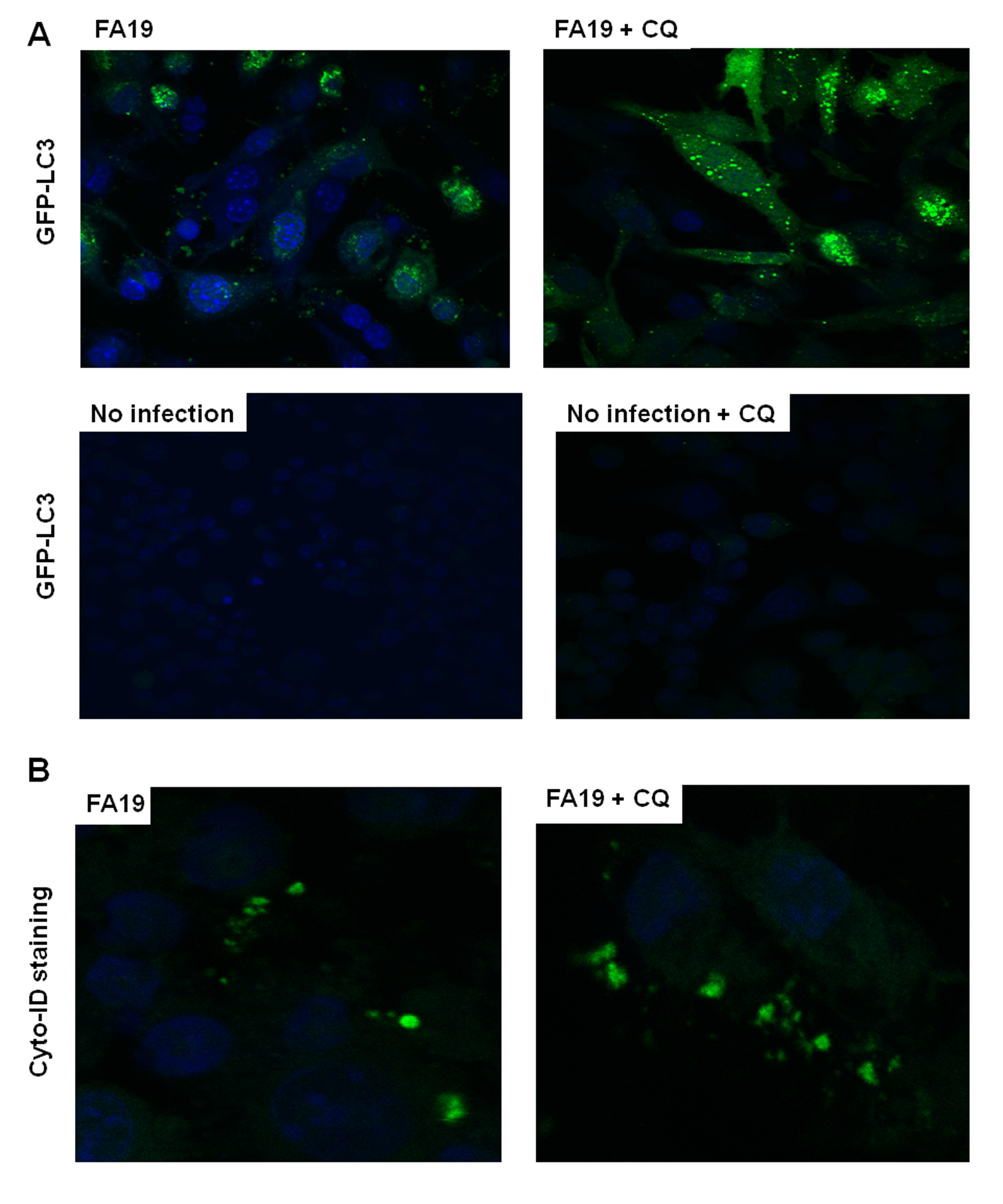

Supplement: S1 Fig — Since Cyto-ID® autophagy probe partitions specifically into the autophagolysosomal vesicles indicating active autophagy flux, chloroquine (CQ) is used as a control for autophagy flux. (A): Representative confocal microscopic images of autophagic puntca formation in GFP-LC3-tagged RAW264 macrophages infected with live Gc strain FA19 at an MOI of 50 with and without CQ (20 μM) treatment. (B): Representative confocal microscopic images of autophagic flux in murine RAW264 macrophages treated with 20 μM CQ, infected with live Gc strain FA19 and stained with Cyto-ID® autophagy probe. (TIF) [file pone.0144347.s001.tif]

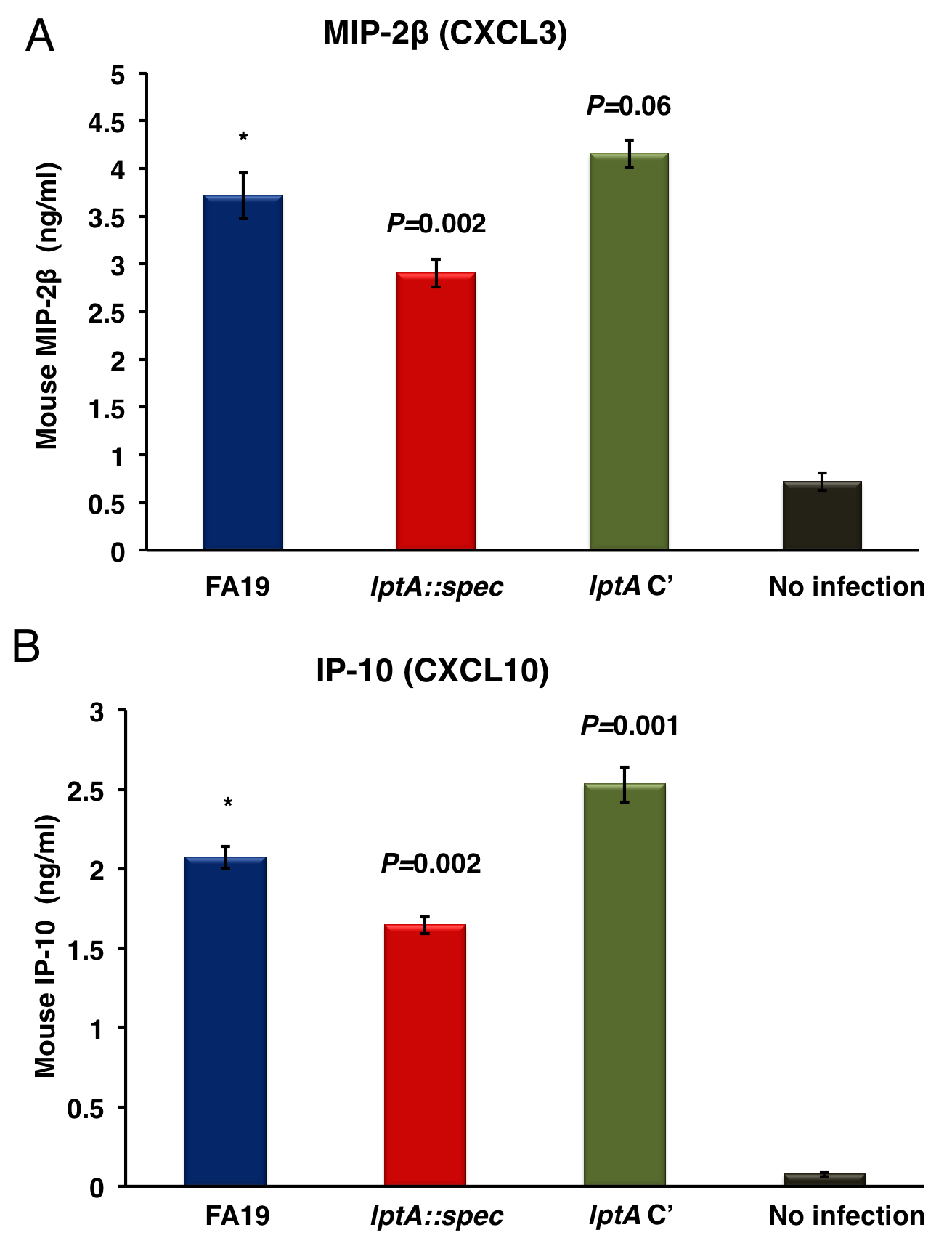

Supplement: S2 Fig — Murine RAW264 macrophages infected with Gc strains at an MOI of 50. Chemokines MIP-2β (A) and IP-10 (B) release from infected macrophages was quantitated by ELISA. Error bars represent the ±SD from the mean chemokine release from three independent biological replicates. p values were calculated using Student t-test in reference to results obtained when cells were infected with the WT FA19 (*) strain. (TIF) [file pone.0144347.s002.tif]
